# Childbearing, Infertility, and Career Trajectories Among Women in Medicine

**DOI:** 10.1001/jamanetworkopen.2023.26192

**Published:** 2023-07-27

**Authors:** Jennifer B. Bakkensen, Kathryn S. Smith, Elaine O. Cheung, Patricia I. Moreno, Kara N. Goldman, Angela K. Lawson, Eve C. Feinberg

**Affiliations:** 1 Division of Reproductive Endocrinology and Infertility, Department of Obstetrics and Gynecology, Northwestern University Feinberg School of Medicine, Chicago, Illinois; 2 Northwestern University Feinberg School of Medicine, Chicago, Illinois; 3 Department of Medical Social Sciences, Northwestern University Feinberg School of Medicine, Chicago, Illinois; 4 Hinge, New York, New York; 5 Department of Public Health Sciences at the University of Miami Miller School of Medicine, Miami, Florida

## Abstract

**Importance:**

Although women are increasingly represented within medicine, gender disparities persist in time to promotion, achievement of academic rank, and appointment to leadership positions, with no narrowing of this gap over time. Career-specific fertility and family building challenges among women physicians may contribute to ongoing disparities and academic attrition.

**Objective:**

To evaluate delayed childbearing and infertility among women in medicine and investigate the extent to which women physicians may alter career trajectories to accommodate family building and parenthood.

**Design, Setting, and Participants:**

This survey study was conducted among women physicians, with surveys distributed through medical society electronic mailing lists (listserves) and social media from March to August 2022.

**Main Outcomes and Measures:**

Baseline demographic information and fertility knowledge were assessed. Descriptive data on delayed childbearing, infertility, use of assisted reproductive technology, and career alterations to accommodate parenthood were collected. Factors associated with timing of pregnancy and family building regret were assessed using Likert-type scales. Group differences in fertility knowledge, delayed childbearing, infertility, and family building regret were evaluated using χ^2^ analyses.

**Results:**

A total of 1056 cisgender women (mean [SD] age, 38.3 [7.7] years) were surveyed across level of training (714 attending physicians [67.6%] and 283 residents or fellows [26.8%]), specialty (408 surgical [38.6%] and 638 nonsurgical [60.4%] specialties), and practice setting (323 academic [45.2%], 263 private [24.9%], and 222 community [21.0%] settings). Among respondents, 1036 individuals [98.1%] resided in the US. Overall, 910 respondents (86.2%) were married or partnered and 690 respondents (65.3%) had children. While 824 physicians (78.0%) correctly identified the age of precipitous fertility decline, 798 individuals (75.6%) reported delaying family building and 389 individuals (36.8%) had experienced infertility. Concerning measures taken to accommodate childbearing or parenthood, 199 women (28.8%) said they had taken extended leave, 171 women (24.8%) said they had chosen a different specialty, 325 women (47.1%) said they had reduced their work hours, 171women (24.8%) said they had changed their practice setting, and 326 women (47.2%) said they had passed up opportunities for career advancement among those with children. Additionally, 30 women with children (4.3%) had left medicine entirely.

**Conclusions and Relevance:**

In this survey study, women physicians reported that career-related pressures influenced the timing of childbearing and led to marked alterations to career trajectories to accommodate family building and parenthood. These findings suggest that fertility and family building concerns among women in medicine may contribute to ongoing gender disparities and attrition and represent a potentially critical area for policy reform and future change.

## Key Points

**Question:**

To what extent do women in medicine alter the timing of pregnancy and modify career trajectories to accommodate parenthood and career?

**Findings:**

This survey study of 1056 women physicians found that despite strong knowledge of age-related fertility decline, three-fourths of women physicians delayed childbearing and more than one-third experienced infertility. Nearly half of women with children reported passing up opportunities for career advancement to accommodate family building and parenthood.

**Meaning:**

In this survey study, women in medicine reported that they delayed pregnancy and altered their career trajectories due to competing priorities of parenthood and career, which may contribute to high rates of infertility and ongoing gender disparities within medicine.

## Teaser

This survey study investigates delays in childbearing and rates of infertility among women physicians.

## Introduction

While women are increasingly represented within medicine,^1^ pervasive gender disparities exist. A landmark 2000 study^2^ found that female US medical school graduates were less likely to be promoted to upper faculty ranks, with a 2020 follow-up study^3^ finding no narrowing of this gap over time. Women are less likely than men to publish in leading medical journals or hold positions on editorial review boards^4,5^ and are less likely to hold academic leadership positions.^6^ According to 2021 data from the Association of American Medical Colleges (AAMC),^6^ women account for 43% of medical school faculty but 22% of department chairs and 22% of medical school deans.

Although reasons for attrition are unclear and likely complex, fertility and family building may be contributing given the duration and intensity of medical training, which coincides with women’s peak reproductive years. Prior research found that women physicians were more likely to delay childbearing and experience infertility compared with nonphysicians.^7,8,9,10,11^ While the decision to delay may be underinformed without full understanding how age is associated with fertility,^12,13^ physicians may postpone childbearing in spite of this knowledge due to insurmountable career-related pressures. The extent to which fertility knowledge may mediate delayed childbearing and infertility is unknown.

While research has focused on the association of a medical career with fertility outcomes, less is known about the association of family building and parenthood with career. A small survey^14^ of physician parents found that women were more likely to have turned down projects or committee participation due to parenting concerns, and a longitudinal cohort study^15^ of medical interns found that women were more likely than men to work part time after completion of training, with family consistently cited as the most influential factor in this decision. There is a need to more thoroughly evaluate family building and parenthood as a factor associated with gender disparities within medicine.

The objectives of this study were to characterize patterns of delayed family building and infertility among women physicians, assess differences in fertility knowledge and their association with delayed family building and infertility, investigate factors associated with family building regret, and (4) investigate the extent to which women in medicine may alter their career to balance parenthood and career.

## Methods

This cross-sectional survey study was approved by the Northwestern University institutional review board. Participants provided informed consent. The study was conducted in accordance with the American Association for Public Opinion Research (AAPOR) reporting guideline.

### Survey Development

**Figure 1.**
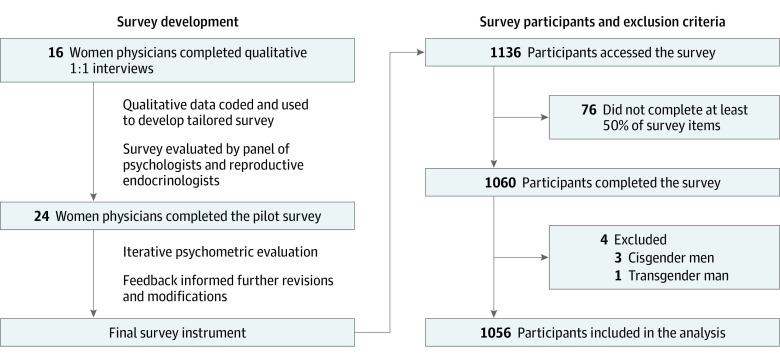
Schematic of Survey Development and Distribution

Survey development was detailed in a prior publication,^16^ as summarized in Figure 1. Briefly, standardized 1:1 interviews exploring perceptions and experiences of fertility, parenthood, and career were conducted among women physicians. Qualitative data were coded in Dedoose version 9.0.107 and used to develop tailored survey items. Questions assessing fertility knowledge were developed with content expertise from reproductive endocrinologists (K.N.G. and E.C.F.) and validated by collaborators with expertise in qualitative and survey research (E.O.C. and P.I.M.). Psychometric evaluation was conducted in a pilot survey among women physicians, with feedback used to inform further revisions and modifications.

### Survey Content

The survey (eAppendix in Supplement 1) assessed demographics, career, work hours, and household responsibilities. To evaluate differences by race and ethnicity, participants were asked to report using options defined by investigators or write-in responses. Race and ethnicity were queried in the same question, and available categories were Asian, Black or African American, Hispanic or Latino, Middle Eastern or North African, Native Hawaiian or Pacific Islander, White, multiracial (available as an option respondents could choose), and other, with the option to provide a write-in response.

Fertility knowledge was assessed through 3 multiple choice items. First, participants were asked over which age range a cisgender woman’s ability to conceive declines most precipitously (25-29, 30-34, or ≥35 years). Then, they were asked to select the likelihood of pregnancy per month with intercourse and cumulative live birth (CLB) per in vitro fertilization (IVF) cycle if a cisgender woman is aged 30 to 34, 35 to 39, 40 to 42, or 43 to 45 years.^17,18^ Beliefs about stress and infertility were queried, and the extent to which participants relied on various sources for fertility information was assessed (“not at all” to “extremely”).

Delayed family building, infertility, and use of IVF were assessed. Experiences with oocyte cryopreservation (OC) or embryo cryopreservation were queried through multiple choice questions. Women were asked about factors they believed influenced the decision to pursue cryopreservation, ranging from “not at all” to “extremely.” Family building and career regret were explored by asking respondents the extent to which they agreed that they would have changed aspects of family planning or career if they could do it over again (“strongly disagree” to “strongly agree”). Finally, participants were asked whether they had taken specific career measures to accommodate childbearing or parenthood.

### Survey Distribution

The survey was distributed through social media March to August 2022. The survey QR code and hyperlink (eFigure 1 in Supplement 1) were distributed through social media, including targeted Facebook physician groups and promotion on Twitter by study investigators; shared through direct emails to residency program directors and alumni associations listed by the AAMC; and circulated through medical society electronic mailing lists (listserves). This included listserves within the American Medical Women’s Association, American Medical Association (AMA), Association of Women Surgeons, American Association of Women Radiologists, Women in Endocrinology, Women in Nephrology, and Women in Ophthalmology.

### Study Participants

Women attending physicians and trainees were invited to participate. Participation was voluntary and uncompensated, and participants provided informed consent. Cisgender men (3 individuals) were excluded. Due to potentially unique circumstances surrounding fertility for transgender men and women, these respondents were also excluded (1 individual). Gay and lesbian women were included. Participants completing 50% of survey items or more were included in the final analysis.

### Statistical Analysis

Descriptive statistics were performed, and χ^2^ analysis with pairwise, Bonferroni-adjusted *z* tests were used to test group differences regarding fertility knowledge (assessed by the correct identification of age at fertility decline), delayed family building (having ever vs never delayed), duration of delay (<3, 3-5, or >5 years), and infertility, as well as differences regarding infertility and specific measures of family building regret. *P* values were 2-sided, and *P* < .05 was regarded as significant. To evaluate whether these outcomes varied by specialty, associations were tested among obstetrics and gynecology physicians vs those in other specialties and in surgical vs nonsurgical specialties. Data were analyzed using SPSS statistical software version 23.0 (IBM).

## Results

### Demographics

A total of 1136 surveys were submitted, and the analysis included responses from 1056 cisgender women (mean [SD] age, 38.3 [7.7] years; 79 Black [7.5%], 42 Hispanic or Latino 42 [4.0%], and 742 White [70.3%]) (Figure 1). Among 1136 individuals who began the survey, 75 individuals did not finish (6.6%). Demographics are summarized in Table 1. Participants were included across level of training (714 attending physicians [67.6%] and 283 residents or fellows [26.8%]), specialty (408 surgical [38.6%] and 638 nonsurgical [60.4%] specialties), and practice setting (323 academic [45.2%], 263 private [24.9%], and 222 community [21.0%] settings). Respondents represented every US state except Nevada, with a small number (20 respondents [1.9%]) residing outside the US; 1036 individuals [98.1%] resided in the US. The most represented specialties included obstetrics and gynecology (321 respondents [30.4%]), internal medicine (189 respondents [17.9%]), and pediatrics (133 respondents [12.6%]). There were 735 respondents in specialties other than obstetrics and gynecology (69.6%). Overall, 910 respondents (86.1%) were married or partnered and 690 respondents (65.3%) had children. Among respondents with children, 218 individuals (31.6%) intended to have additional children, and among 363 respondents without children, 290 individuals (79.9%) intended to have children in the future. A comparison of characteristics between survey respondents and US women physicians is shown in the eTable in Supplement 1. Overall, survey respondents were younger than US women physicians (mean age, 51.5 years).

**Table 1.** Respondent Characteristics

Characteristic	Respondents, No. (%) (N = 1056)^a^
Age, mean (SD), y (n = 930)^b^	38.3 (7.7)
Age, y	
≤34	331 (31.3)
35-40	304 (28.8)
≥41	295 (27.9)
Missing	126 (11.9)
Race and ethnicity	
Asian	142 (13.4)
Black or African American	79 (7.5)
Hispanic or Latino	42 (4.0)
Middle Eastern or North African	13 (1.2)
Multiracial^c^	27 (2.4)
Native Hawaiian or Pacific Islander	1 (0.1)
White	742 (70.3)
Other^d^	5 (0.5)
Prefer not to answer	5 (0.5)
Residence	
US region	
Northeast	181 (17.1)
Midwest	346 (32.8)
South	342 (32.4)
West	167 (15.8)
Outside the US	20 (1.9)
Years since medical school graduation, mean (SD)	11.3 ± 8.0
Current position	
Attending physician	714 (67.6)
Resident or fellow	283 (26.8)
Other^e^	59 (5.6)
Practice type among attending physicians (n = 714)	
Academic	323 (45.2)
Private or community	328 (45.9)
Other^f^	63 (8.8)
Specialty type	
Surgical	408 (38.6)
Nonsurgical	638 (60.4)
Missing	10 (0.9)
Marital status	
Married or partnered	910 (86.1)
Single	123 (11.6)
Divorced	19 (1.8)
Other	4 (0.4)
Sexual orientation	
Heterosexual	978 (92.6)
Gay or lesbian	27 (2.6)
Bisexual	42 (4.0)
Other^g^	5 (0.5)
Prefer not to answer	4 (0.4)
Have children	
Yes	690 (65.3)
No	363 (34.4)
Prefer not to answer	3 (0.3)
Among respondents with children (n = 690)	
No. of children, mean (SD)	2.0 (0.9)
Intend to have additional children	218 (31.6)
Among respondents with no children, intend to have children in the future (n = 363)	290 (79.9)
Household income, $	
<100 000	130 (12.3)
100 000-250 000	279 (26.4)
250 000-500 000	431 (40.8)
≥500 000	201 (19.0)
Prefer not to answer	15 (1.4)
Current work, h/wk	
0-19	36 (3.5)
20-39	150 (14.2)
40-59	543 (51.4)
≥60	316 (29.9)
Other^h^	11 (1.0)
Among married or partnered, household maintenance roles assigned, mean (SD), % (n = 910)	
Self	49.2 (20.7)
Partner	40.5 (20.5)
Paid family member or other	7.7 (14.9)
Unpaid family member or other	2.2 (9.3)
Missing	0.3 (4.9)
Among married or partnered with children, family maintenance roles assigned, mean(SD), % (n = 668)	
Self	58.3 (27.0)
Partner	28.4 (22.3)
Paid family member or other	8.3 (18.0)
Unpaid family member or other	3.3 (11.3)
Missing	1.7 (12.8)

^a^ Numbers may not add to 100% due to rounding.

^b^ Among 930 respondents who provided their age.

^c^ Multiracial was 1 of the choices participants could select if they felt it best applied to them. If not, they could choose other with a write-in response.

^d^ Write-in responses included Ashkenazi Jewish, European, Hispanic Black, Southeast Asian, and White British.

^e^ Responses included retired attending physician, private practice physician, and volunteer teaching faculty.

^f^ Responses included hospital-based practice, Veterans Affairs or military medicine, student health, and hospital-based outpatient practice.

^g^ Responses included queer.

^h^ Responses included retired, furloughed, unemployed, disabled, and irregular or hospitalist hours.

Nearly 30% of respondents (369 individuals [29.9%]) worked 60 or more hours per week, whereas among married or partnered women, 130 respondents (14.3%) reported that their partner worked 60 or more hours per week. Respondents reported bearing most household maintenance roles (eg, cleaning, buying groceries, cooking, and doing laundry), with married or partnered women ascribing a mean (SD) of 49.2% (20.7%) of these roles to themselves vs 40.5% (20.5%) to their spouses or partners. Among 660 married or partnered women with children, respondents similarly reported taking on a greater proportion of family maintenance roles (eg, childcare, eldercare, health care appointments, and school forms), assigning a mean of 58.4% (27.0%) to themselves vs 28.2% (22.3%) to their partners (Table 1).

### Fertility Knowledge

**Figure 2.**
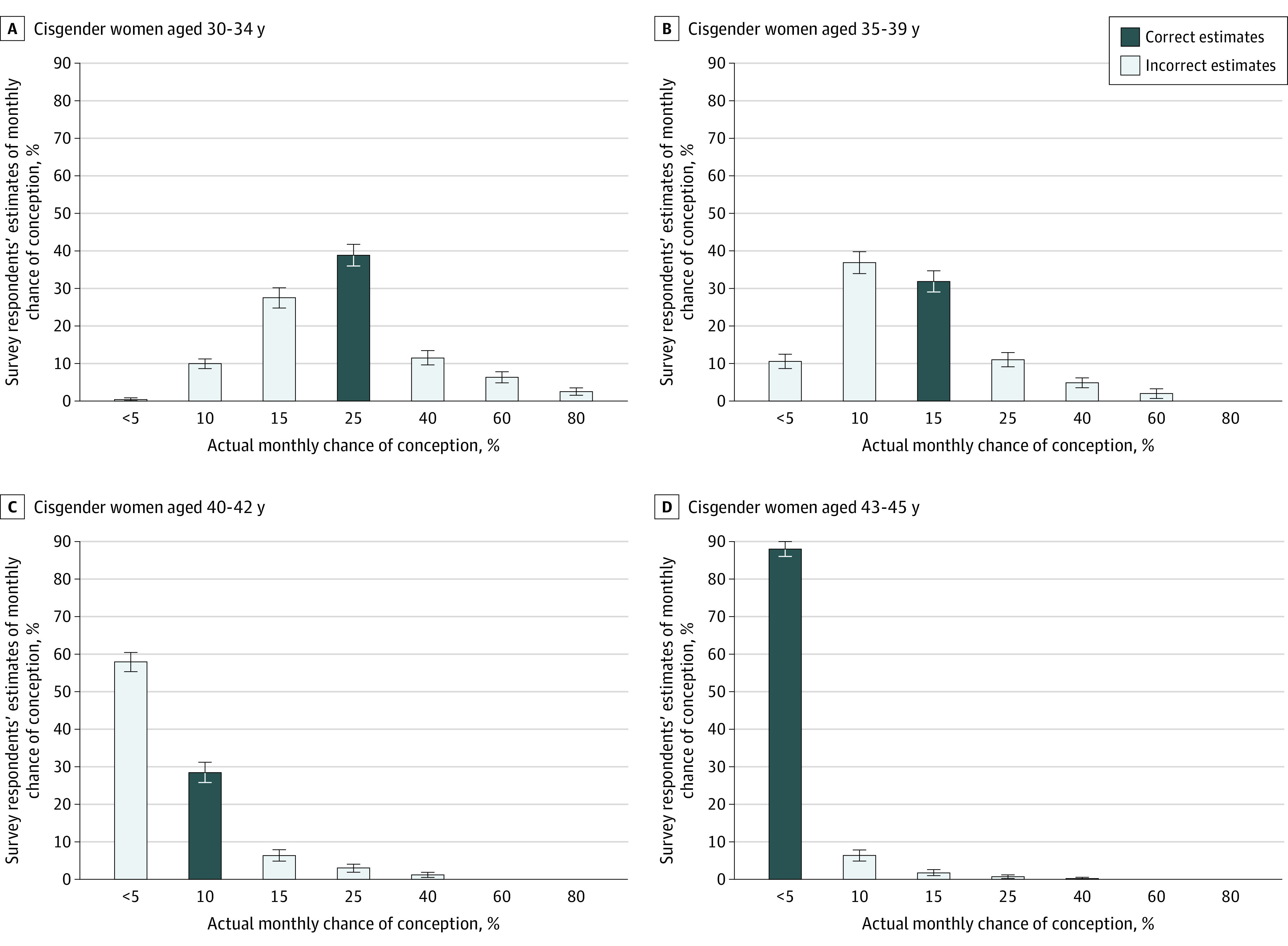
Survey Respondent Estimation of Monthly Chance of Conception by Age Error bars indicate 95% CIs.

Among all respondents, 824 individuals (78.0%) correctly identified the age of fertility decline of 35 years or older. Most respondents correctly or nearly correctly identified approximate monthly chance of conception by age, with incorrect answers tending to underestimate vs overestimate fertility in most age groups (Figure 2). The most frequently selected likelihood of conception for each group decreased with increasing maternal age, with 930 respondents (88.1%) correctly estimating the chance of conception to be less than 5% among women aged 43 to 45 years (Figure 2).

Estimates for CLB with IVF were more dispersed, again with a tendency to underestimate vs overestimate success rates within each age group (eFigure 2 in Supplement 1). While most respondents (648 individuals [61.4%]) correctly identified a likelihood of CLB of less than 5% for women aged 43 to 45 years, 408 respondents (38.6%) overestimated the likelihood of success within this age group (eFigure 2 in Supplement 1).

Obstetrics and gynecology physicians demonstrated higher fertility knowledge vs other physicians, with 274 respondents (85.4%) vs 567 respondents (77.1%) correctly identifying the age of fertility decline (*P* = .002). Experiences of colleagues and personal experiences were most frequently cited as sources of information (268 participants [25.4%] and 292 participants [27.7%] responded “very much” or “extremely,” respectively), while 261 respondents (24.7%) said they relied on formal medical education “very much” or “extremely.”

Most respondents (758 individuals [71.8%]) said they believed that stress causes infertility at least “a little.” While obstetrics and gynecology physicians were less likely than physicians from other specialties to harbor this belief (196 respondents [61.0%] vs 561 respondents [76.3%]; *P* < .001), most obstetrics and gynecology physicians endorsed at least some causal role.

### Delayed Family Building

Most respondents (988 individuals [93.6%]) reported concern regarding the length of training and family planning, with 798 respondents (75.6%) reporting delaying family building due to medical training or specialty choice and 213 respondents (20.2%) reporting not delaying (Table 2). Results did not vary by specialty. Of respondents who delayed, 182 individuals (22.8%) had delayed more than 5 years. Reasons for delay included lack of schedule flexibility or time, stress, financial strain, and concern about burdening colleagues. Among respondents who delayed, 105 individuals (13.1%) had concerns about lack of support from leadership “very much” or “extremely.” There was no association between fertility knowledge and delay or between specialty and delay.

**Table 2.** Family Planning and Infertility Questions

Question	Respondents, No. (%) (N = 1056)^a^
Have you ever felt concerned about how the length of your medical training would impact your family planning?	
Not at all	68 (6.4)
A little bit	246 (23.3)
Moderately	368 (34.8)
Very	243 (23.0)
Extremely	131 (12.4)
Missing	0
Did you delay having children because of your medical training or career?	
Yes, in the past	583 (55.2)
Yes, currently	215 (20.4)
No	213 (20.2)
Missing	45 (4.3)
If you delayed, how long did you delay (or plan to delay) having children? (n = 798)	
<3 y	367 (46.0)
3-5 y	248 (31.1)
>5 y	182 (22.8)
Missing	1 (0.1)
Have you ever considered egg/embryo freezing for fertility preservation?	
Yes	448 (42.4)
No	606 (57.4)
Missing	2 (0.2)
Is egg/embryo freezing covered by your health insurance?	
Yes	105 (9.9)
No	440 (41.7)
Don’t know	509 (48.2)
Missing	2 (0.2)
If you learned that egg/embryo freezing was covered by your health plan, would you use it?	
Yes	402 (38.1)
No	385 (36.5)
Unsure	266 (25.2)
Missing	3 (0.3)
Did you freeze your eggs/embryos?	
Yes	121 (11.5)
No	897 (84.9)
Other^b^	38 (3.6)
If you froze eggs/embryos, at what age did you freeze? mean (SD), y (n = 121)	34.4 (3.0)
Have you ever experienced infertility?	
Yes	389 (36.8)
No	428 (40.5)
Have not tried to conceive	228 (21.6)
Prefer not to answer	11 (1.1)
If you experienced infertility, have you used IVF to conceive? (n = 389)	
Yes	200 (51.4)
No	186 (47.8)
Missing	3 (0.8)
If you used IVF, at what age did you undergo IVF? mean (SD), y (n = 200)	34.5 (3.7)

Abbreviation: IVF, in vitro fertilization.

^a^ Numbers may not add to 100% due to rounding.

^b^ Responses included “Yes, but as part of IVF/fertility treatment”; “Not yet, but currently in process”; “Strongly considering in the next year”; and “Attempted to freeze embryos but none were genetically normal so have none frozen.”

### Oocyte or Embryo Cryopreservation

While 448 respondents (42.4%) considered oocyte or embryo cryopreservation, 121 respondents (11.5%) had completed treatment (Table 2), among whom 37 respondents (30.6%) had insurance that covered these procedures. Overall, 105 respondents had insurance coverage for these procedures (9.9%). Respondents cited colleagues and family and friends as the most influential sources of knowledge. Among all respondents, 331 women (31.3%) said they relied on colleagues and 262 women (24.8%) said they relied on family and friends as at least moderately as sources of information. Women cited age, financial cost, and insurance coverage as the most important factors in the decision to pursue the procedure. Among women who completed oocyte or embryo cryopreservation, 100 individuals (82.6%) rated age, 85 individuals (70.2%) rated financial cost, and 80 individuals (65.3%) rated insurance coverage as at least moderately important.

### Infertility

Among all respondents, 389 individuals (36.8%) experienced infertility, among whom 200 individuals (51.4%) had used IVF (18.9% of all respondents) (Table 2). Women who delayed were more likely to experience infertility than those who did not delay (420 respondents [52.6%] vs 69 respondents [32.4%]; *P* < .001), with higher rates of infertility among those who had delayed more than 5 years (117 women [64.3%]) or 248 women who delayed 3 to 5 years (154 women [62.1%]) vs 367 women who delayed less than 3 years (156 women [42.5%]) (*P* < .001). There was no association between knowledge or specialty and infertility.

### Family Building Regret

When asked in retrospect what they would do differently, 483 respondents [45.7%] said they would have conceived earlier, 473 respondents [44.8%] said they would have reduced work hours, 410 respondents [38.8%] said they would have taken extended leave, and 300 respondents [28.4%] said they would have pursued OC. Women with infertility were more likely to agree that they would have tried to conceive earlier vs 656 women who did not experience infertility or had not tried to conceive (282 women [72.5%] vs 222 women [22.8%]; *P* < .001) or pursued OC (160 women [41.1%] vs 96 women [14.7%]; *P* < .001).

### Career Accommodations

**Figure 3.**
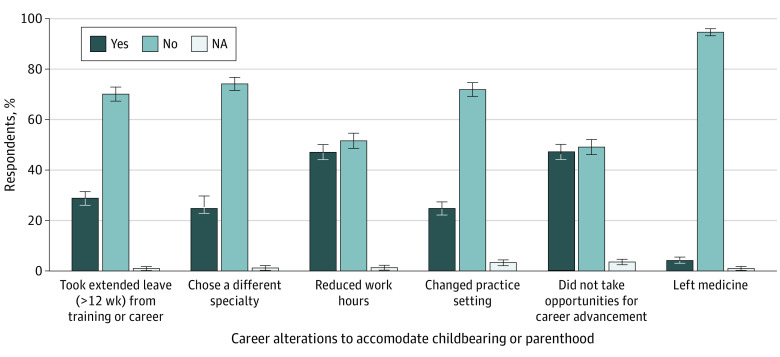
Career Alterations Among Women Physicians With Children to Accommodate Pregnancy or Parenthood Error bars indicate 95% CIs.

Among respondents with children, 199 women (28.8%) had taken an extended leave from training or career, 171 women (24.8%) had chosen a different specialty, 325 women (47.1%) had reduced work hours, and 171 women (24.8%) had changed practice settings. Nearly half of respondents (326 women [47.2%]) reported passing up opportunities for career advancement to accommodate childbearing or parenthood, and 30 women (4.3%) had left medicine (Figure 3).

## Discussion

In this survey study, women physicians cited significant career-related pressures as influencing the timing of childbearing and reported marked career alterations to accommodate parenthood. To our knowledge, this is one of the largest studies to evaluate fertility and family building among women physicians and the first survey to evaluate the association of family building and parenthood with career. While we did not limit the survey to US physicians, more than 98% of respondents reported residing in the US, suggesting that these results may be most representative of US-based physicians.

Our findings contribute to a growing body of literature characterizing unique family building challenges among women physicians. The prevalence of delayed childbearing among women in medicine was identified in a 2016 survey^10^ and supported by a 2021 retrospective cohort study^7^ that found the mean age at first birth to be 32 years among physicians vs 27 years among nonphysicians. More than three-quarters of female physicians in our survey reported delaying childbearing due to medical training or career. This figure is striking in light of the well-documented decline in female fertility with age.^19,20^ Although some studies have suggested low fertility knowledge among physicians,^12,13^ our findings agreed with prior data suggesting an understanding of the decrease in fertility with age and showed that respondents had a tendency to underestimate vs overestimate fertility.^10^ Importantly, knowledge was not associated with likelihood of delayed childbearing or duration of delay. Given these results, the factors cited as most influential regarding timing of childbearing (ie, lack of schedule flexibility and time, stress, and financial strain) may represent barriers to earlier pregnancy despite this knowledge.

Alarmingly, 36.8% of respondents endorsed a personal history of infertility, among whom more than half required IVF to conceive. In contrast, 6% to 19% of women in the US general population have infertility and 12.2% have used fertility services.^21,22^ Although our sample may not be representative, the prevalence was similar to prior estimates.^10^ While delayed childbearing may be a key factor associated with this rate of infertility, other career-specific risk factors should be considered. Despite respondents’ overwhelmingly predominant belief that psychological stress may have a causal role in infertility, conclusive evidence is lacking whether this causal role exists^23,24,25,26,27^ and interventions designed to reduce psychological stress have failed to demonstrate benefit.^28,29^ However, lifestyle factors, such as shift work and poor sleep quality, have been proposed as factors associated with impaired fertility and pregnancy outcomes^30,31^ and warrant further evaluation.

Furthermore, our results highlight the degree to which women in medicine altered their careers to accommodate pregnancy and parenthood. More than one-quarter of respondents reported taking an extended leave of 12 weeks or greater, notably higher than the 14.9% of US medical schools currently offering faculty 12 weeks or more of fully paid maternity leave.^32^ In support of single-center data,^14^ nearly half of women with children in our study reported passing up opportunities for career advancement and reducing work hours to accommodate parenthood. Results from a 2019 prospective study^15^ of early-career physicians suggested that these accommodations may be more prevalent among women vs men physicians and may begin soon after completion of training; within 6 years of training, 22.6% of women physicians worked part time vs 3.6% of men physicians, and 77.5% of those working part time cited family as the most influential factor in this decision. The emergence of this gap at such an early and foundational time in physician careers suggests that it is plausible that fertility and family building concerns may contribute to gender inequities in medicine.

Collectively, these data highlight a need to support women physicians in balancing family and career. Paid parental leave is not federally mandated in the US,^33^ and nearly half of top-ranked medical schools do not provide paid parental leave for birth (42%) or nonbirth (44%) faculty.^32^ Encouragingly, a recent Accreditation Council for Graduate Medical Education mandate specifies that sponsoring institutions must have policies including 6 or more paid weeks of parental leave.^34^ While this falls short of the 12 weeks recommended by the American Academy of Pediatrics on the basis of parental and infant benefits,^35^ it represents an important step. Policies must be accompanied by cultural shifts to counter concerns that taking leave damages professional reputation^36,37^ and leads to loss of career opportunities.^38,39^ Furthermore, policies must provide leave for birth and nonbirth parents to counter norms of women shouldering a disproportionate burden of childcare and household responsibilities. Additionally, access to the full spectrum of fertility care must be expanded. In addition to offering insurance benefits and clinical flexibility to the nearly 20% of women physicians who use IVF to conceive, awareness of and access to fertility preservation services should be offered to those desiring greater flexibility in family planning. In this survey study, 11.5% of women underwent oocyte or embryo cryopreservation, and less than 10% had insurance coverage of the procedures. Although the AMA has supported resident physician access to oocyte and embryo cryopreservation,^40^ buy-in at the institutional level is needed to implement these benefits and expand coverage to medical students and faculty. Until that happens, access to fertility preservation may be unattainable for medical trainees and junior faculty during the years it is most likely to be effective.^41^

### Limitations

This study has several limitations. The chief limitation was our inability to calculate response rate due to the unknown number of individuals who received the survey hyperlink. While the number of individuals who began but did not finish the survey was known (76 individuals), most respondents provided insufficient information on which to compare nonrespondents with respondents. Survey respondents were younger vs all US women physicians, indicating some response bias. It is possible that women who personally struggled with infertility were more likely to complete the survey, thereby inflating the prevalence within our sample. However, respondents spanned all levels of training and practice patterns across all regions of the US. The most common specialties included those with predominately women populations, such as obstetrics and gynecology and pediatrics, which may explain the relative overrepresentation within our cohort. Additionally, the reliance on self-report introduced the possibility of recall bias. While we cannot eliminate the possibility of bias, the consistency of findings between qualitative^16^ and this quantitative data suggests that while point estimates may vary, prevalence was high and warrants attention. To our knowledge, this cohort represents one of the largest on this topic to date, but no sample size calculation was performed.

Notably, the decision to focus on women was made due to previous data suggesting that women physicians were more likely to delay childbearing,^9^ experience infertility,^9^ and alter their careers for family reasons.^14,15^ Trends among cisgender men physicians and transgender physicians warrant further investigation. Furthermore, the relatively small number of gay and lesbian respondents precluded meaningful evaluation of the potentially distinct challenges and concerns within this population, which should be the subject of future research.

Additionally, given that fewer than 2% of respondents resided outside the US, results may be less applicable to physicians from other countries. While the prolonged duration of medical training and experience of age-related fertility decline are universal, national policies and societal norms may vary and represent an important line of inquiry for future study.

## Conclusions

Findings from this survey of women physicians suggest that career-related pressures may be associated with the timing of pregnancy and may contribute to significant rates of infertility despite adequate fertility knowledge and that family building and parenthood may be associated with alterations in the career trajectory of women in medicine. These findings highlight a need for ongoing research into the reasons underlying delayed family building and infertility within this population and a need for targeted support to address the disparate and discriminatory experiences of women physicians. While there is clearly more work to be done, these data shed light on potentially critical areas for policy reform and future change.

## Notes

Supplement 1. **eTable.** Characteristics of Respondents and All Us Women Physicians From Published Sources **eFigure 1.** Infographic and QR Code Used for Participant Recruitment on Social Media **eFigure 2.** Respondents Estimation of Cumulative Likelihood of Live Birth Per In Vitro Fertilization Cycle by Age **eAppendix.** Survey Instrument

Supplement 2.
Data Sharing Statement
