# Supplementary material for: Childbearing, Infertility, and Career Trajectories Among Women in Medicine
Source: JAMA Netw Open. 2023 Jul 27;6(7):e2326192. doi: 10.1001/jamanetworkopen.2023.26192 (PMC10375303; doi:10.1001/jamanetworkopen.2023.26192)
Supplement: Supplement 1. — eTable. Characteristics of Respondents and All Us Women Physicians From Published Sources eFigure 1. Infographic and QR Code Used for Participant Recruitment on Social Media eFigure 2. Respondents Estimation of Cumulative Likelihood of Live Birth Per In Vitro Fertilization Cycle by Age eAppendix. Survey Instrument [file jamanetwopen-e2326192-s001.pdf]

# Supplemental Online Content

Bakkensen JB, Smith KS, Cheung EO, et al. Childbearing, infertility, and career trajectories among women in medicine. *JAMA Netw Open*. 2023;6(7):e2326192. doi:10.1001/jamanetworkopen.2023.26192

**eTable.** Characteristics of Respondents and All Us Women Physicians From Published Sources
**eFigure 1.** Infographic and QR Code Used for Participant Recruitment on Social Media
**eFigure 2.** Respondents Estimation of Cumulative Likelihood of Live Birth Per In Vitro Fertilization Cycle by Age
**eAppendix.** Survey Instrument

This supplemental material has been provided by the authors to give readers additional information about their work.

© 2023 Bakkensen JB et al. *JAMA Network Open.*

**eTable 1.** Characteristics reported among survey respondents (n = 1,056) and among all US women physicians according to published sources[a,b]

| Characteristic | Survey respondents | US women physicians |
|---|---|---|
| *Age (years)* | 38.3 | 51.5 [c] |
| *Race/Ethnicity* | | |
| Asian | 13.4% | 19.8% [d] |
| Black/African American | 7.5% | 5.8% [d] |
| Hispanic/Latino | 4.0% | 6.7% [d] |
| Native Hawaiian/Pacific Islander | 0.1% | 0.3% [d] |
| White | 70.3% | 65.2% [d] |
| Other | 4.7% | 2.1% [d] |
| *Current position* | | |
| Attending | 67.6% | 84.2% [e] |
| Resident/fellow | 26.8% | 15.8% [e] |
| Other/missing | 5.6% | |
| *Specialty type* | | |
| Surgical | 38.6% | 14.4% [f] |
| Non-surgical | 60.4% | 85.6% [f] |
| Other/missing | 10 (0.9%) | |
| *Sexual Orientation* | | |
| Heterosexual | 92.6% | 94.5% [g] |
| Gay or lesbian | 2.6% | 1.7% [g] |
| Bisexual | 4.0% | 3.8% [g] |
| Other/missing | 0.9% | |

[a] Data presented as mean or % as appropriate.
[b] Numbers may not add to 100% due to rounding.
[c] Average age of all active physicians in US workforce. Source: FSMB Census of Licensed Physicians in the United States, 2020
[d] Among all active physicians in the US workforce for whom race and ethnicity was known. AAMC Diversity in Medicine: Facts and figures 2019.
[e] Among all US physicians. Source: AAMC report on residents, 2022 and AAMC physician specialty report, 2022.
[f] Source: AAMC physician specialty report, 2022.
[g] Source: Mori et. al., *JAMA Netw Open*, 2021; doi: 10.1001/jamanetworkopen.2021.26983

© 2023 Bakkensen JB et al. *JAMA Network Open.*

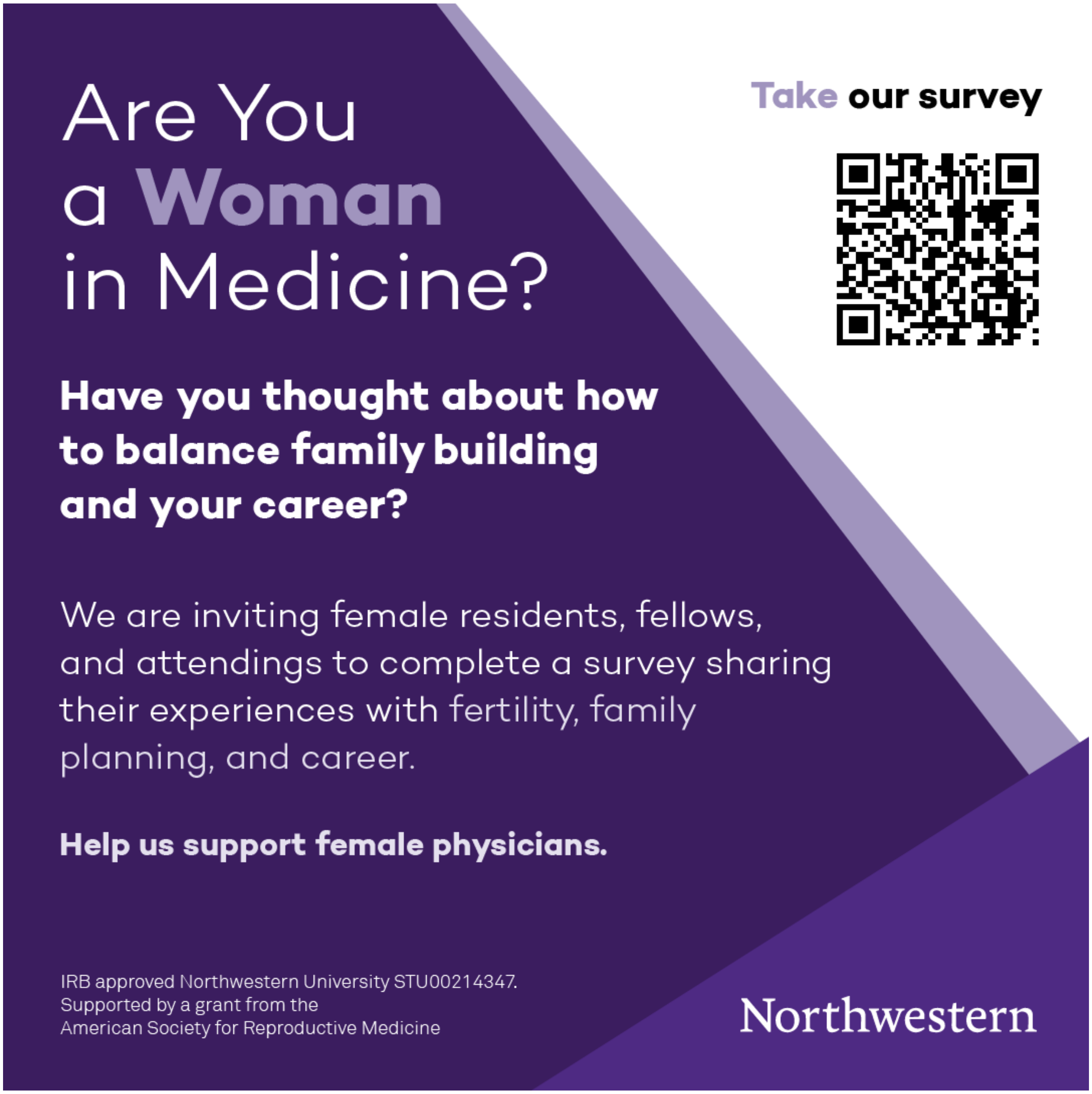

**eFigure 1.** Infographic and QR code used for participant recruitment on social media.

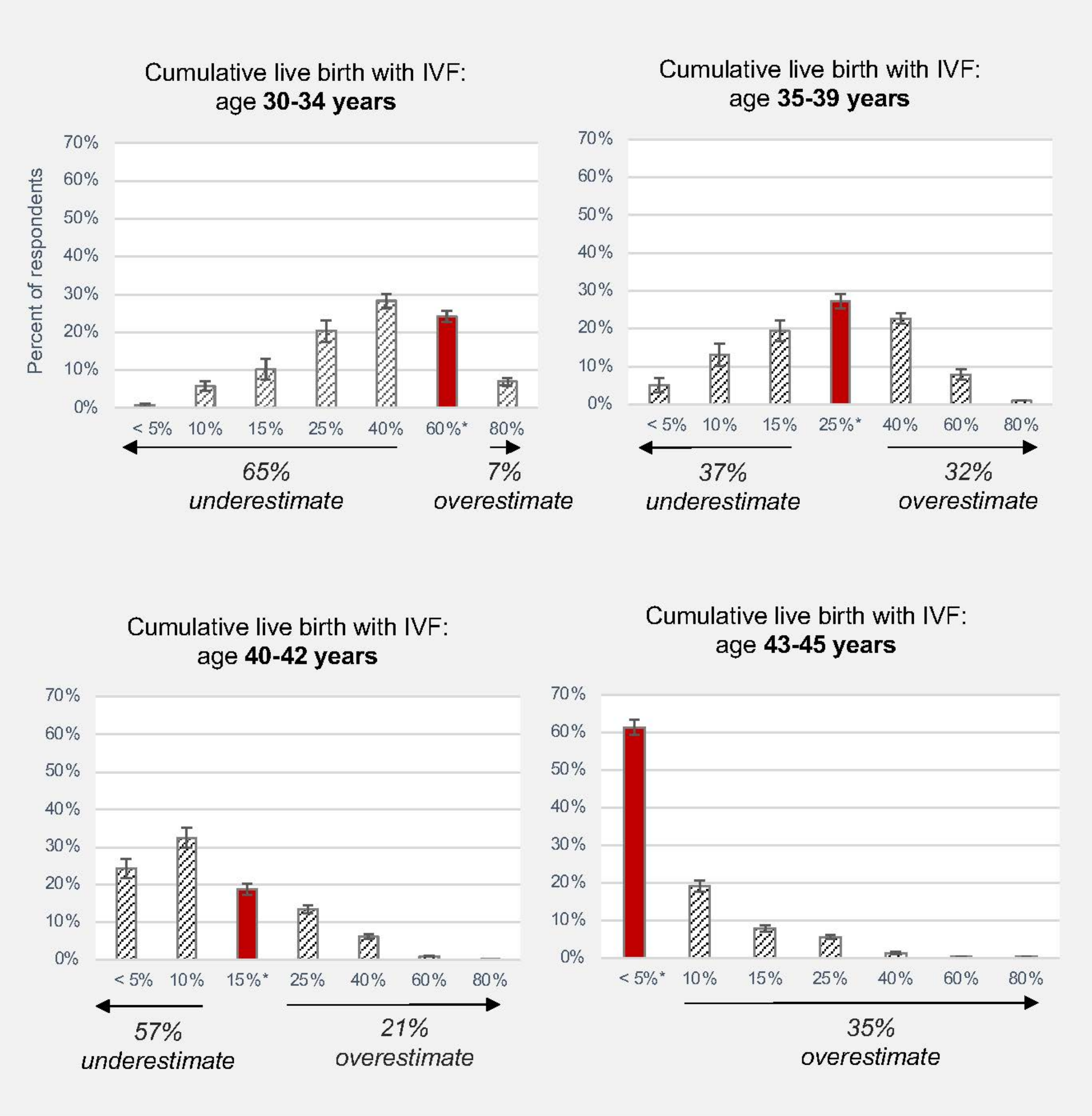


**eFigure 2.** Survey respondents' estimation of the cumulative likelihood of live birth per IVF cycle by cisgender woman's age. The most correct estimate for each age range is indicated in red.

© 2023 Bakkensen JB et al. *JAMA Network Open.*

**eAppendix.**

---

Start of Block: Welcome

Intro **Title of Research Study:** Fertility and Family Planning Experiences of Women in Medicine
**Principal Investigator:** Eve Feinberg, MD
**Supported By:** This research is supported by a research grant from the American Society for Reproductive Medicine.
**Conflict of Interest Disclosure:** The researchers have no conflicts of interest to disclose.

**Key Information about this research study:** The following is a short summary of the study's purpose and procedure to help you decide whether to take part in the study. More detailed information will be included in the remainder of this document. The purpose of this study is to better understand the fertility and family planning experiences of female physicians, and how these experiences may influence the career paths that women take. Participants will be asked about their fertility and family planning experiences, their knowledge and understanding of the role of age in the efficiency and success of fertility preservation, and how fertility concerns impacts both personal and professional decision-making. If you consent to participate in this study, you will be asked to complete an online questionnaire which will take approximately 10-15 minutes to complete. The primary potential risk of participation is discomfort, as some of the questions are sensitive in nature and may ask you to recall difficult and/or stressful experiences. However, you can skip any question you do not wish to answer or exit the survey at any point. The main benefit of taking part in this study is helping to shape the discourse around fertility and fertility preservation among women in medicine.
**Why am I being asked to take part in this research study?** We are asking you to take part in this research study because you are a woman over the age of 18 years of age who has graduated from medical school. This research includes only individuals who choose to take part, so please take your time to make your decision about participating. If you have any questions, you may contact the researchers.
**How many people will be in this study?** We expect up to 2500 people will be in this research study. However, the actual number may vary depending on the number of responses received.

**What should I know about participating in a research study?** · Whether or not you take part is up to you. · You can choose not to take part. · You can agree to take part and later change your mind. · Your decision will not be held against you. · You can ask all the questions you want before you decide. · You do not have to answer any question you do not want to answer.

**What happens if I say, "Yes, I want to be in this research"?** If you consent to participate in this study, you will be asked to complete a 10–15-minute online questionnaire using Northwestern's secure online survey software (Qualtrics). The survey will be conducted entirely online.

**Will being in this study help me in any way?** We cannot promise any benefits to you or others from your taking part in this research. However, our research team wants to hear from

you, so that we may learn what policies and changes may best support female physicians like you in the future.

**Is there any way being in this study could be bad for me?** There is a risk of discomfort, as some of the questions may ask you to recall difficult experiences. You can skip any question you do not wish to answer or exit the survey at any point. A possible risk for any research is that confidentiality could be compromised – that is, that people outside the study might get hold of confidential study information. We will do everything we can to minimize this risk, as described in more detail later in this form.

**What happens if I do not want to be in this research, or I change my mind later?** Participation in research is voluntary. You can decide to participate or not to participate. You can decide not to participate in this research, or you can start and then decide to leave the research at any time, and it will not be held against you. To do so, simply exit the survey.

**How will the researchers protect my information?** All survey responses will be collected through an online survey-collection program called Qualtrics. Only the study investigators will have access to the data on Qualtrics. Information submitted by participants will be stored in encrypted form on Northwestern computers on the Feinberg School of Medicine's Departmental Server, which is a secure, password-protected serve. All data will be analyzed and presented without identifying information.

**Who will have access to the information collected during this research study?** Efforts will be made to limit the use and disclosure of your personal information, including research study records, to people who have a need to review this information. We cannot promise complete secrecy.

There are reasons why information about you may be used or seen by other people beyond the research team during or after this study. Examples include: · University officials, government officials, study funders, auditors, and the Institutional Review Board may need access to the study information to make sure the study is done in a safe and appropriate manner.

This survey is being hosted by Qualtrics and involves a secure connection. Terms of service, addressing confidentiality, may be viewed at http://www.qualtrics.com/research-suite/. You will be identified only by a unique subject number. All information will be kept on a password protected computer only accessible by the research team. The results of the research study may be published.

**How might the information collected in this study be shared in the future?** We will keep the information we collect about you during this research study for study recordkeeping and for future use in research projects.

De-identified data from this study may be shared with the research community, with journals in which study results are published, and with databases and data repositories used for research. We will remove or code any personal information that could directly identify you before the study data are shared. Despite these measures, we cannot guarantee anonymity of your personal data.

**Will I be paid or given anything for taking part in this study?** There is no payment or reimbursement for participating in this study.

**Who can I talk to?** If you have questions, concerns, or complaints talk to the Principal

© 2023 Bakkensen JB et al. *JAMA Network Open.*

Investigator Eve Feinberg, MD, at (312) 926-8244
This research has been reviewed and approved by an Institutional Review Board ("IRB") – an IRB is a committee that protects the rights of people who participate in research studies. You may contact the IRB by phone at (312) 503-9338 or by email at irb@northwestern.edu if:
· Your questions, concerns, or complaints are not being answered by the research team.
· You cannot reach the research team. · You want to talk to someone besides the research team. · You have questions about your rights as a research participant. · You want to get information or provide input about this research. If you want a copy of this consent for your records, you can print it from the screen.

---

Consent If you wish to participate, please click the "I Agree" button and you will be taken to the survey. If you do not wish to participate in this study, please select "I Disagree" or select X in the corner of your browser.

○ I Agree (1)

○ I Disagree (2)

End of Block: Welcome

Start of Block: Welcome & demographics

Age What is your date of birth (mm/dd/yyyy)?

________________________________________________________________

---

© 2023 Bakkensen JB et al. *JAMA Network Open.*

Gender What is your gender identity?

- Male (1)
- Female (2)
- Non-binary (3)
- Genderfluid (4)
- Not listed (please specify) (5) ________________________________________________
- Prefer not to answer (6)

---

Assigned Sex What was your assigned sex at birth?

- Male (1)
- Female (2)
- Prefer not to answer (3)

---

Sex Orientation What is your sexual orientation?

- Heterosexual (or straight) (1)
- Gay or lesbian (2)
- Bisexual (3)
- Not listed (please specify) (4) ________________________________________________
- Prefer not to answer (5)

---

© 2023 Bakkensen JB et al. *JAMA Network Open.*

Race With which race/ethnicity do you identify?

- ○ White (1)
- ○ Black or African American (2)
- ○ Asian (3)
- ○ American Indian or Alaska Native (4)
- ○ Hispanic or Latino (5)
- ○ Native Hawaiian or Other Pacific Islander (6)
- ○ Middle Eastern or North African (7)
- ○ Multiracial (8)
- ○ Not listed (please specify) (9) ________________________________________________
- ○ Prefer not to answer (10)

---

Income Which of these categories best describes your total combined household income for the past 12 months?

- ○ < $99K (1)
- ○ $100K - $250K (2)
- ○ $250K - $500K (3)
- ○ > $500K (4)
- ○ Don't know (5)
- ○ Prefer not to answer (6)

© 2023 Bakkensen JB et al. *JAMA Network Open.*

Relationship What is your current relationship status?

- ◯ Married/Partnered (1)
- ◯ Single (2)
- ◯ Divorced/Separated (3)
- ◯ Widowed (4)
- ◯ Not Listed (please specify) (5) ________________________________________________

HoursWorked How many hours a week do you currently work?

- ◯ 1 to 19 hours (1)
- ◯ 20 to 39 hours (2)
- ◯ 40 to 59 hours (3)
- ◯ > 60 hours/week (4)
- ◯ Not employed (5)
- ◯ Furloughed (6)
- ◯ Retired (7)
- ◯ Not Listed (please specify) (8) ________________________________________________

Page Break

© 2023 Bakkensen JB et al. *JAMA Network Open.*

*Display This Question:*

*If What is your current relationship status? = Married/Partnered*

PartnerWork How many hours a week does your spouse/partner work?

- ◯ 1 to 19 hours (1)
- ◯ 20 to 39 hours (2)
- ◯ 40 to 59 hours (3)
- ◯ > 60 hours/week (4)
- ◯ Not employed (5)
- ◯ Furloughed (6)
- ◯ Retired (7)
- ◯ Not Listed (please specify) (8) ________________________________________________

Page Break

© 2023 Bakkensen JB et al. *JAMA Network Open.*

HouseholdMaintenance On a scale from 0-100%, please allocate how the responsibilities for maintaining your household and family life are distributed in your household for **household maintenance (e.g., cleaning, groceries, cooking, laundry).** If the situation is not applicable to you, please allocate 100% to the "Not Applicable" option.
You : _______ (1)
Spouse/Partner : _______ (2)
Paid Family Member/Other : _______ (3)
Unpaid Family Member/Other : _______ (4)
Not Applicable : _______ (5)
Total : ________

FamilyMaintenance On a scale from 0-100%, please allocate how the responsibilities for maintaining your household and family life are distributed in your household for **family maintenance (e.g., childcare, eldercare, healthcare appointments, school forms).** If the situation is not applicable to you, please allocate 100% to the "Not Applicable" option.
You : _______ (1)
Spouse/Partner : _______ (2)
Paid Family Member/Other : _______ (3)
Unpaid Family Member/Other : _______ (4)
Not Applicable : _______ (5)
Total : ________

**End of Block: Welcome & demographics**

**Start of Block: State**

Q1 In which state do you currently reside?

▼ Alabama (1) ... I do not reside in the United States (53)

**End of Block: State**

**Start of Block: Medical Career/Training**

MedSchool In what year did you graduate medical school?

________________________________________________________________

© 2023 Bakkensen JB et al. *JAMA Network Open.*

PracticingMedicine Are you currently practicing medicine?

- Yes (1)
- No (2)

Position What is your current position?

- Resident (1)
- Fellow (2)
- Attending Physician (3)
- Not Listed (please specify) (4) ________________________________________________

Specialty In what specialty/specialties have you trained?

________________________________________________________________

Page Break

*Display This Question:*

*If What is your current position? = Attending Physician*

CurrentPractice What type of practice setting do you currently work in? Please check all that apply.

- ○ Academic practice (1)
- ○ Private practice (2)
- ○ Community practice (3)
- ○ Not listed (please specify) (4) ________________________________________________

*Display This Question:*

*If What is your current position? = Resident*

*Or What is your current position? = Fellow*

PlannedPractice What type of practice do you plan to go into? Please check all that apply.

- ▢ Academic practice (1)
- ▢ Private practice (2)
- ▢ Community practice (3)
- ▢ Not listed (please specify) (4) ________________________________________________

Page Break

© 2023 Bakkensen JB et al. *JAMA Network Open.*

Burnout_1 I feel burned out from my work.

- Never (0)
- A few times a year (1)
- Once a month or less (2)
- A few times a month (3)
- Once a week (4)
- A few times a week (5)
- Every day (6)

---

Burnout_2 I have become more callous toward people since I took this job.

- Never (0)
- A few times a year (1)
- Once a month or less (2)
- A few times a month (3)
- Once a week (4)
- A few times a week (5)
- Every day (6)

End of Block: Medical Career/Training

---

Start of Block: Medical History

© 2023 Bakkensen JB et al. *JAMA Network Open.*

Children Do you have children (e.g., biological, adopted, step, or foster children)?

- Yes (1)
- No (2)
- Prefer not to answer (3)

Page Break

© 2023 Bakkensen JB et al. *JAMA Network Open.*

*Display This Question:*

*If Do you have children (e.g., biological, adopted, step, or foster children)? = Yes*

X→

Bio_children How many biological children do you have?

- 0 (0)
- 1 (1)
- 2 (2)
- 3 (3)
- 4 (4)
- 5+ (5)

*Display This Question:*

*If Do you have children (e.g., biological, adopted, step, or foster children)? = Yes*

X→

Nonbio_children How many adopted, step, or foster children do you have?

- 0 (0)
- 1 (1)
- 2 (2)
- 3 (3)
- 4 (4)
- 5+ (5)

Page Break

© 2023 Bakkensen JB et al. *JAMA Network Open.*

*Display This Question:*

*If Do you have children (e.g., biological, adopted, step, or foster children)? = Yes*

Additional_Children Do you intend to have additional children in the future? (e.g., biological, adopted, step, or foster)?

- Yes (1)
- No (2)
- Undecided (3)
- Prefer not to answer (4)

Page Break

© 2023 Bakkensen JB et al. *JAMA Network Open.*

*Display This Question:*

*If Do you intend to have additional children in the future? (e.g., biological, adopted, step, or fos... = Yes*

AddBioChildren How many additional biological children do you plan to have in the future?

- 0 (0)
- 1 (1)
- 2 (2)
- 3 (3)
- 4 (4)
- 5+ (5)

---

*Display This Question:*

*If Do you intend to have additional children in the future? (e.g., biological, adopted, step, or fos... = Yes*

AddNonBioChildren How many additional non-biological children (e.g., adoption, fostering) do you plan to have in the future?

- 0 (0)
- 1 (1)
- 2 (2)
- 3 (3)
- 4 (4)
- 5+ (5)

---

Page Break

*Display This Question:*

*If Do you have children (e.g., biological, adopted, step, or foster children)? = No*

IntendFutureChildren Do you intend to have children in the future (e.g., biological, adopted, step, or foster)?

- Yes (1)
- No (2)
- Undecided (3)
- Prefer not to answer (4)

---

*Display This Question:*

*If Do you intend to have children in the future (e.g., biological, adopted, step, or foster)? = Yes*

IntendBioChildren How many biological children do you plan to have in the future?

- 0 (0)
- 1 (1)
- 2 (2)
- 3 (3)
- 4 (4)
- 5+ (5)

---

*Display This Question:*

*If Do you intend to have children in the future (e.g., biological, adopted, step, or foster)? = Yes*

© 2023 Bakkensen JB et al. *JAMA Network Open.*

IntendNonBioChildren How many non-biological children (e.g., adoption, fostering) do you plan to have in the future?

- 0 (0)
- 1 (1)
- 2 (2)
- 3 (3)
- 4 (4)
- 5+ (5)

Page Break

© 2023 Bakkensen JB et al. *JAMA Network Open.*

*Display This Question:*

*If What was your assigned sex at birth? = Female*

X→

Pregnancy How many times have you been pregnant (if any)?

- ○ 0 (0)
- ○ 1 (1)
- ○ 2 (2)
- ○ 3 (3)
- ○ 4 (4)
- ○ 5+ (5)

Page Break

© 2023 Bakkensen JB et al. *JAMA Network Open.*

*Display This Question:*

*If How many times have you been pregnant (if any)? != 0*

*And What was your assigned sex at birth? = Female*

X→ X→

PregnancyOutcomes What were the outcome(s) of the pregnancy/pregnancies? (Please select all that apply).

- ▢ Live born (1)
- ▢ Miscarriage (2)
- ▢ Termination (3)
- ▢ Stillbirth (5)
- ▢ Not Listed (please specify) (4) ________________________________________________

End of Block: Medical History

---

Start of Block: Influence of training/career on family planning or vice versa

*Display This Question:*

*If How many times have you been pregnant (if any)? != 0*

*And What was your assigned sex at birth? = Female*

X→

PregnantImpactCareer Do you think your pregnancy outcome(s) was impacted by your medical training or career?

- ○ Yes (1)
- ○ No (2)
- ○ Undecided (3)
- ○ Prefer not to answer (4)

Page Break

© 2023 Bakkensen JB et al. *JAMA Network Open.*

*Display This Question:*

*If Do you think your pregnancy outcome(s) was impacted by your medical training or career? = Yes*

PregnancyOutcomeTEXT Please specify how your pregnancy outcome(s) may have been impacted by your medical training or career:

________________________________________________________________

________________________________________________________________

________________________________________________________________

________________________________________________________________

________________________________________________________________

Page Break

© 2023 Bakkensen JB et al. *JAMA Network Open.*

DelayChildbearing Did you delay having children because of your medical training or career? (Please select all that apply).

- ☐ Yes, I have delayed in the past (1)
- ☐ Yes, I am currently still delaying (2)
- ☐ No (3)
- ☐ Other (please specify) (4) ________________________________________________
- ☐ Prefer not to answer (5)

Page Break

© 2023 Bakkensen JB et al. *JAMA Network Open.*

*Display This Question:*

*If Did you delay having children because of your medical training or career? (Please select all that... = Yes, I have delayed in the past*

*Or Did you delay having children because of your medical training or career? (Please select all that... = Yes, I am currently still delaying*

X→ X→

DelayDuration How long did you delay (or plan to delay) having children?

- ◯ 0 to 3 years (1)
- ◯ 3 to 5 years (2)
- ◯ 5+ years (3)

---

Page Break

© 2023 Bakkensen JB et al. *JAMA Network Open.*

© 2023 Bakkensen JB et al. *JAMA Network Open.*

Factors_Timing Did the following factors influence your decisions about the timing of having children?

© 2023 Bakkensen JB et al. *JAMA Network Open.*

| | Not at all (1) | A little bit (2) | Moderately (3) | Very Much (4) | Extremely (5) |
|---|---|---|---|---|---|
| Lack of time (Lack of time) | ○ | ○ | ○ | ○ | ○ |
| Lack of flexibility in schedule (Lack of flexibility in schedule) | ○ | ○ | ○ | ○ | ○ |
| Financial strain (Financial strain) | ○ | ○ | ○ | ○ | ○ |
| Lack of romantic partner (Lack of romantic partner) | ○ | ○ | ○ | ○ | ○ |
| Lack of support from colleagues (Lack of support from colleagues) | ○ | ○ | ○ | ○ | ○ |
| Concern about burdening colleagues with extra work (Concern about burdening colleagues) | ○ | ○ | ○ | ○ | ○ |
| Reputational concerns/stigma (e.g., being perceived as less committed to career) (Reputational concerns/stigma) | ○ | ○ | ○ | ○ | ○ |
| Lack of support from leadership (e.g., chair, division chief, practice manager) (Lack of support from leadership) | ○ | ○ | ○ | ○ | ○ |

© 2023 Bakkensen JB et al. *JAMA Network Open.*

| | | | | | |
|---|---|---|---|---|---|
| Lack of social support nearby (e.g., extended family, friends) (Lack of social support nearby ) | ○ | ○ | ○ | ○ | ○ |
| Stress (Stress) | ○ | ○ | ○ | ○ | ○ |
| Not ready for children (Not ready for children) | ○ | ○ | ○ | ○ | ○ |

Page Break

© 2023 Bakkensen JB et al. *JAMA Network Open.*

X→

Training_Impact_Fam Have you ever felt concerned about how the length of your medical training would impact your family planning?

- ◯ Not at all concerned (1)
- ◯ A little bit concerned (2)
- ◯ Moderately concerned (3)
- ◯ Very concerned (4)
- ◯ Extremely concerned (5)

---

Page Break

© 2023 Bakkensen JB et al. *JAMA Network Open.*

X→ X→

Family_Impact_Career Have you done the following to accommodate childbearing or parenthood?

| | Yes (1) | No (2) | Not Applicable (3) |
|---|---|---|---|
| Taken an extended leave (over 12 weeks) from your medical training or career to accommodate childbearing or parenthood (Extended Leave) | ○ | ○ | ○ |
| Chosen a different specialty or career to accommodate childbearing or parenthood (Different Specialty) | ○ | ○ | ○ |
| Reduced your work hours to accommodate childbearing or parenthood (Reduce Work Hours) | ○ | ○ | ○ |
| Left medicine to accommodate childbearing or parenthood (Left Medicine) | ○ | ○ | ○ |
| Changed the setting of your work (e.g., academic vs. private practice) (Change Practice Setting) | ○ | ○ | ○ |
| Not taken opportunities for career advancement (Not taken opportunities) | ○ | ○ | ○ |

End of Block: Influence of training/career on family planning or vice versa

Start of Block: Egg Freezing Questions

© 2023 Bakkensen JB et al. *JAMA Network Open.*

*Display This Question:*

*If What was your assigned sex at birth? = Female*

eggfreezing Have you ever considered egg/embryo freezing for fertility preservation?

- Yes (1)
- No (2)
- Prefer not to answer (3)

Page Break

© 2023 Bakkensen JB et al. *JAMA Network Open.*

*Display This Question:*

*If Have you ever considered egg/embryo freezing for fertility preservation? = Yes*

SeekConsultation Did you seek consultation for egg/embryo freezing at a fertility center?

- Yes (1)
- No (2)
- Other (please specify) (3) ________________________________________________
- Prefer not to answer (4)

Page Break

© 2023 Bakkensen JB et al. *JAMA Network Open.*

*Display This Question:*

*If Have you ever considered egg/embryo freezing for fertility preservation? = Yes*

FreezeEggs Did you freeze your eggs/embryos?

- Yes (1)
- No (2)
- Other (please specify) (3) ________________________________________________
- Prefer not to answer (4)

Page Break

© 2023 Bakkensen JB et al. *JAMA Network Open.*

*Display This Question:*

*If Did you freeze your eggs/embryos? = Yes*

AgeFreeze At what age did you freeze eggs/embryos?

________________________________________________________________

Page Break

© 2023 Bakkensen JB et al. *JAMA Network Open.*

*Display This Question:*

*If Did you freeze your eggs/embryos? = No*

*Or Did you freeze your eggs/embryos? = Other (please specify)*

IntendtoFreeze Do you plan to freeze your eggs/embryos in the future?

- Yes (1)
- No (2)
- Don't Know (3)
- Other (please specify) (4) ________________________________________________
- Prefer not to answer (5)

Page Break

© 2023 Bakkensen JB et al. *JAMA Network Open.*

*Display This Question:*

*If Do you plan to freeze your eggs/embryos in the future? = Yes*

AgeFreeze At what age do you plan to freeze your eggs/embryos?

________________________________________________________________

Page Break

© 2023 Bakkensen JB et al. *JAMA Network Open.*

InsuranceEggFreezing Is egg/embryo freezing covered by your health insurance?

- Yes (1)
- No (2)
- Don't know (3)

Cost_EggFreezing Are you aware of the approximate out-of-pocket cost that is associated with freezing your eggs/embryos?

- Yes (1)
- No (2)

Use_Insurance_Egg If you learned that egg/embryo freezing was covered by your health plan, would you use it?

- Yes (1)
- No (2)
- Not sure (3)

© 2023 Bakkensen JB et al. *JAMA Network Open.*

Willing2PayEggFreeze If egg/embryo freezing was not covered by your health plan, how much would you be willing to pay out of pocket to freeze your eggs/embryos?

- ◯ $0  (1)
- ◯ $1 to $999  (2)
- ◯ $1000 to $4,999  (3)
- ◯ $5000 to $9,999  (4)
- ◯ $10,000 to $19,999  (5)
- ◯ $20,000+  (6)

Page Break

© 2023 Bakkensen JB et al. *JAMA Network Open.*

*Display This Question:*

*If Have you ever considered egg/embryo freezing for fertility preservation? = Yes*

Factors_EggFreeze Did the following factors influence your initial consideration of freezing eggs/embryos or your decision about whether or not to go through the process of freezing eggs/embryos?

| | Not at all (1) | A little bit (2) | Moderately (3) | Very Much (4) | Extremely (5) | Not Applicable (6) |
|---|---|---|---|---|---|---|
| Relationship status (Relationship status) | ○ | ○ | ○ | ○ | ○ | ○ |
| Age (Age) | ○ | ○ | ○ | ○ | ○ | ○ |
| Financial cost (Financial cost) | ○ | ○ | ○ | ○ | ○ | ○ |
| Insurance coverage (Insurance coverage) | ○ | ○ | ○ | ○ | ○ | ○ |
| Time commitment (Time commitment) | ○ | ○ | ○ | ○ | ○ | ○ |
| Potential risks associated with procedure (Potential risks associated with procedure) | ○ | ○ | ○ | ○ | ○ | ○ |
| Efficacy/likelihood of success (Effectiveness/likelihood of success) | ○ | ○ | ○ | ○ | ○ | ○ |
| Burden (physical and emotional) associated with procedure (Burden) | ○ | ○ | ○ | ○ | ○ | ○ |
| Ethical/Religious concerns (Ethics) | ○ | ○ | ○ | ○ | ○ | ○ |

Page Break

© 2023 Bakkensen JB et al. *JAMA Network Open.*

*Display This Question:*

*If What was your assigned sex at birth? = Female*

Infertility Have you ever experienced infertility?

- Yes (1)
- No (2)
- Have not tried to conceive (3)
- Not applicable (4)
- Prefer not to answer (5)

---

*Display This Question:*

*If Have you ever experienced infertility? = Yes*

IVF Have you ever used in vitro fertilization (IVF) for conception?

- Yes (1)
- No (2)
- Not applicable (4)
- Prefer not to answer (5)

---

Page Break

© 2023 Bakkensen JB et al. *JAMA Network Open.*

*Display This Question:*

*If Have you ever used in vitro fertilization (IVF) for conception? = Yes*

Age_IVF  At what age did you undergo in vitro fertilization (IVF)?

________________________________________________________________

**End of Block: Egg Freezing Questions**

---

**Start of Block: Fertility knowledge**

FertilityKnowledge Thank you for sharing your personal experiences. We are also interested in assessing female physicians' knowledge of fertility and fertility preservation and the sources that female physicians rely on to learn this knowledge. We hope this information may help inform our understanding of how to better educate medical trainees on the role of age in fertility and fertility preservation.

Page Break

© 2023 Bakkensen JB et al. *JAMA Network Open.*

FertilityDecline Over which age range does a cisgender woman’s ability to get pregnant decline most precipitously?

- 25 to 29 years old (1)
- 30 to 34 years old (2)
- Over 35 years old (3)

Page Break

© 2023 Bakkensen JB et al. *JAMA Network Open.*

Page Break

© 2023 Bakkensen JB et al. *JAMA Network Open.*

Likelihood_Pregnancy What is the likelihood of pregnancy per month if a cisgender female is attempting conception and is:

| | | 10% (2) | 15% (3) | 25% (4) | 40% (5) | 60% (6) | 80% (7) |
|---|---|---|---|---|---|---|---|
| 30 to 34 years old (30 to 34 years old) | ○ | ○ | ○ | ○ | ○ | ○ | ○ |
| 35 to 39 years old (35 to 39 years old) | ○ | ○ | ○ | ○ | ○ | ○ | ○ |
| 40 to 43 years old (40 to 44 years old) | ○ | ○ | ○ | ○ | ○ | ○ | ○ |
| 43 to 45 years old (43 to 45 years old) | ○ | ○ | ○ | ○ | ○ | ○ | ○ |

Page Break

© 2023 Bakkensen JB et al. *JAMA Network Open.*

Likelihood_IVF What is the likelihood of live birth for a cisgender female using her own eggs in one IVF cycle if the female is:

| | | 10% (2) | 15% (3) | 25% (4) | 40% (5) | 60% (6) | 80% (7) |
|---|---|---|---|---|---|---|---|
| 30 to 34 years old (30 to 34 years old) | ○ | ○ | ○ | ○ | ○ | ○ | ○ |
| 35 to 39 years old (35 to 39 years old) | ○ | ○ | ○ | ○ | ○ | ○ | ○ |
| 40 to 43 years old (40 to 43 years old) | ○ | ○ | ○ | ○ | ○ | ○ | ○ |
| 43 to 45 years old (43 to 45 years old) | ○ | ○ | ○ | ○ | ○ | ○ | ○ |

Page Break

© 2023 Bakkensen JB et al. *JAMA Network Open.*

Q77 How much do you think psychological stress causes:

| | Not at all (1) | A little (2) | A moderate amount (3) | A lot (4) |
|---|---|---|---|---|
| Infertility (1) | ○ | ○ | ○ | ○ |
| Pregnancy loss (2) | ○ | ○ | ○ | ○ |
| Poor pregnancy outcomes (3) | ○ | ○ | ○ | ○ |
| Decreased success in fertility treatment(s) (4) | ○ | ○ | ○ | ○ |

Q78 How much do you think psychological stress causes:

| | Not at all (1) | A little (2) | A moderate amount (3) | A lot (4) |
|---|---|---|---|---|
| Infertility (1) | ○ | ○ | ○ | ○ |
| Pregnancy loss (2) | ○ | ○ | ○ | ○ |
| Poor pregnancy outcomes (3) | ○ | ○ | ○ | ○ |
| Decreased success in fertility treatment(s) (4) | ○ | ○ | ○ | ○ |

End of Block: Fertility knowledge

Start of Block: Informational Sources for the Role of Age in Fertility and Egg Freezing

© 2023 Bakkensen JB et al. *JAMA Network Open.*

Sources_Fertility To what extent have you relied on the following sources to learn about the **role of age in fertility**?

© 2023 Bakkensen JB et al. *JAMA Network Open.*

| | Not at all (1) | A little bit (2) | Moderately (3) | Very Much (4) | Extremely (5) |
|---|---|---|---|---|---|
| Formal education in medical school (Formal education in medical school) | ○ | ○ | ○ | ○ | ○ |
| Your own healthcare provider (e.g., OBGYN, PCP) (Your own healthcare provider) | ○ | ○ | ○ | ○ | ○ |
| Your own personal experiences (Your own personal experiences) | ○ | ○ | ○ | ○ | ○ |
| Experiences of family and friends (Experiences of family and friends) | ○ | ○ | ○ | ○ | ○ |
| Experiences of patients (Experiences of patients) | ○ | ○ | ○ | ○ | ○ |
| Experiences of female physician colleagues (Experiences of female physician colleagues) | ○ | ○ | ○ | ○ | ○ |
| Internet (Internet) | ○ | ○ | ○ | ○ | ○ |
| News outlets (News outlets) | ○ | ○ | ○ | ○ | ○ |

© 2023 Bakkensen JB et al. *JAMA Network Open.*

| | | | | | |
|---|---|---|---|---|---|
| Television/film (Television/film) | ○ | ○ | ○ | ○ | ○ |
| Individual posts from social media (e.g., Instagram, Twitter, Facebook) (Individual posts from social media ) | ○ | ○ | ○ | ○ | ○ |
| Educational content from social media (e.g., Instagram, Twitter, Facebook) (Educational content from social media ) | ○ | ○ | ○ | ○ | ○ |

Page Break

© 2023 Bakkensen JB et al. *JAMA Network Open.*

© 2023 Bakkensen JB et al. *JAMA Network Open.*

Sources_eggfreezing To what extent have you relied on the following sources to learn about **elective egg freezing?**

© 2023 Bakkensen JB et al. *JAMA Network Open.*

| | Not at all (1) | A little bit (2) | Moderately (3) | Very Much (4) | Extremely (5) |
|---|---|---|---|---|---|
| Formal education in medical school (Formal education in medical school) | ○ | ○ | ○ | ○ | ○ |
| Your own healthcare provider (e.g., OBGYN, PCP) (Your own healthcare provider) | ○ | ○ | ○ | ○ | ○ |
| Your own personal experiences (Your own personal experiences) | ○ | ○ | ○ | ○ | ○ |
| Experiences of family and friends (Experiences of family and friends) | ○ | ○ | ○ | ○ | ○ |
| Experiences of patients (Experiences of patients) | ○ | ○ | ○ | ○ | ○ |
| Experiences of female physician colleagues (Experiences of female physician colleagues) | ○ | ○ | ○ | ○ | ○ |
| Internet (Internet) | ○ | ○ | ○ | ○ | ○ |
| News outlets (News outlets) | ○ | ○ | ○ | ○ | ○ |

© 2023 Bakkensen JB et al. *JAMA Network Open.*

| | | | | | |
|---|---|---|---|---|---|
| Television/film (Television/film) | ○ | ○ | ○ | ○ | ○ |
| Individual posts from social media (e.g., Instagram, Twitter, Facebook) (Individual posts from social media ) | ○ | ○ | ○ | ○ | ○ |
| Educational content from social media (e.g., Instagram, Twitter, Facebook) (Educational content from social media ) | ○ | ○ | ○ | ○ | ○ |

Page Break

© 2023 Bakkensen JB et al. *JAMA Network Open.*

© 2023 Bakkensen JB et al. *JAMA Network Open.*

DoDifferently If you could do it over again, would you do have done the following differently regarding your family planning and career decisions?

© 2023 Bakkensen JB et al. *JAMA Network Open.*

| | Strongly Disagree (1) | Disagree (2) | Neutral (3) | Agree (4) | Strongly Agree (5) | Not Applicable (6) |
|---|---|---|---|---|---|---|
| Attempt to conceive earlier (Attempt to conceive earlier) | ○ | ○ | ○ | ○ | ○ | ○ |
| Start trying to conceive later (Start trying to conceive later) | ○ | ○ | ○ | ○ | ○ | ○ |
| Freeze your eggs (Freeze your eggs to extend fertility) | ○ | ○ | ○ | ○ | ○ | ○ |
| Take an extended leave (over 12 weeks) from your medical training or career to accommodate childbearing or parenthood (Take an extended leave ) | ○ | ○ | ○ | ○ | ○ | ○ |
| Choose a different specialty or career to accommodate childbearing or parenthood (Choose a different specialty or career) | ○ | ○ | ○ | ○ | ○ | ○ |

© 2023 Bakkensen JB et al. *JAMA Network Open.*

| | | | | | | |
|---|---|---|---|---|---|---|
| Reduce your work hours to accommodate childbearing or parenthood (Reduce your work hours) | ○ | ○ | ○ | ○ | ○ | ○ |
| Leave medicine to accommodate childbearing or parenthood (Leave medicine) | ○ | ○ | ○ | ○ | ○ | ○ |

**End of Block: Informational Sources for the Role of Age in Fertility and Egg Freezing**

**Start of Block: Final thoughts**

Final Thoughts If there is anything else that you would like to share about your experiences with fertility, family planning, and your career, please use the space here. All answers will be fully anonymized upon receipt, so please feel free to share openly.

________________________________________________________________

________________________________________________________________

________________________________________________________________

________________________________________________________________

________________________________________________________________

Page Break

© 2023 Bakkensen JB et al. *JAMA Network Open.*

ThankYou Thank you so much for participating in our study!

End of Block: Final thoughts

© 2023 Bakkensen JB et al. *JAMA Network Open.*
