# Supplementary material for: Childbearing, Infertility, and Career Trajectories Among Women in Medicine
Source: JAMA Netw Open. 2023 Jul 27;6(7):e2326192. doi: 10.1001/jamanetworkopen.2023.26192 (PMC10375303; doi:10.1001/jamanetworkopen.2023.26192)
Supplement: Supplement 2. — Data Sharing Statement [file jamanetwopen-e2326192-s002.pdf]

Data Sharing Statement

Bakkensen. Childbearing, Infertility, and Career Trajectories Among Women in Medicine. *JAMA Netw Open*. Published online July 27, 2023. doi:10.1001/jamanetworkopen.2023.26192

Data
Data available: Yes
Data types: Deidentified participant data
How to access data: jennifer.bakkensen@northwestern.edu **When available:** With publication

Supporting Documents
Document types: None

Additional Information
Who can access the data: Researchers whose proposed use of the data has been approved
Types of analyses: For any purpose
Mechanisms of data availability: With investigator support after approval of a proposal.
